# A Proteomics Study on the Mechanism of Nutmeg-Induced Hepatotoxicity

**DOI:** 10.3390/molecules26061748

**Published:** 2021-03-20

**Authors:** Wei Xia, Zhipeng Cao, Xiaoyu Zhang, Lina Gao

**Affiliations:** School of Forensic Medicine, China Medical University, Shenyang 110122, China; lessenziale123@outlook.com (W.X.); zpcao@cmu.edu.cn (Z.C.); AjaxZhang123@126.com (X.Z.)

**Keywords:** nutmeg, hepatotoxicity, proteomics, oxidative stress, CYP450s, lipid peroxidation

## Abstract

Nutmeg is a traditional spice and medicinal plant with a variety of pharmacological activities. However, nutmeg abuse due to its hallucinogenic characteristics and poisoning cases are frequently reported. Our previous metabolomics study proved the hepatotoxicity of nutmeg and demonstrated that high-dose nutmeg can affect the synthesis and secretion of bile acids and cause oxidative stress. In order to further investigate the hepatotoxicity of nutmeg, normal saline, 1 g/kg, 4 g/kg nutmeg were administrated to male Kunming mice by intragastrical gavage for 7 days. Histopathological investigation of liver tissue, proteomics and biochemical analysis were employed to explore the mechanism of liver damage caused by nutmeg. The results showed that a high-dose (4 g/kg) of nutmeg can cause significant increased level of CYP450s and depletion of antioxidants, resulting in obvious oxidative stress damage and lipid metabolism disorders; but this change was not observed in low-dose group (1 g/kg). In addition, the increased level of malondialdehyde and decreased level of glutathione peroxidase were found after nutmeg exposure. Therefore, the present study reasonably speculates that nutmeg exposure may lead to liver injury through oxidative stress and the degree of this damage is related to the exposure dose.

## 1. Introduction

Nutmeg, *Myristica fragrans Houtt*, has been a common household spice since the Middle Ages and was introduced into Europe by Arabs in the middle of the twelfth century [1]. In addition, to being used as a spice, Middle Eastern physicians discovered the medical value of nutmeg in the ninth century [2]. In folk medicine, nutmeg is used to treat gastrointestinal diseases, psychiatric disorders, respiratory diseases, skin diseases, plague, cholera, musculoskeletal and arthritic disorders; it is also used as an embalming agent, abortifacient and anesthetic [3,4,5,6]. Nutmeg contains many volatile substances, mainly including myristicin, elemicin, safrole, eugenol, isoeugenol, methyl eugenol and geraniol terpenes [5,7,8]. According to pharmacological studies, nutmeg has multiple biological effects, such as antioxidant, antibacterial, anti-inflammatory, hypolipidemic and hypoglycemic and antidepression, and as well, it can be used in the treatment of obesity and type II diabetes [9,10,11,12].

However, nutmeg, which can cause hallucinations at high doses (3–5 g) for human beings, has been used as a hallucinogen since the Crusades [13,14]. The psychoactive effects of nutmeg may be associated with myristicin, elemicin and safrole. Beyond its psychoactive effects, nutmeg can induce injury to the liver, kidney, spleen, heart, medial geniculate body and superior colliculus [4,15]. There have been a considerable number of reported cases of nutmeg poisoning [1,3,7,16,17,18]. Myristicin, one of the main components of nutmeg, can lead to fatty degeneration of the liver after ingestion in a large amount [19]. So far, however, the hepatotoxicity of nutmeg is still unclear. In our previous study, metabolomics was employed to prove the hepatotoxicity of nutmeg and demonstrated that high-dose nutmeg can affect the synthesis and secretion of bile acids and cause oxidative stress, which is one of the most important and common mechanisms of tissue and organ damage [20].

Recently, proteomics has been widely used in life science, such as cell biology, neurobiology and so on. Proteomics offers complementary information to genomics and transcriptomics and is essential for molecular level understanding of the complex biochemical process [21], which helps us cognize the structure and functions of a particular protein and understand the mechanism of damage. Further, it is crucial for early diagnosis, prognosis and to monitor the disease development [22]. Based on the advantages of proteomics, the present study used histopathology, proteomics and biochemical analysis to explore the role of oxidative stress in nutmeg abuse-induced hepatotoxicity.

## 2. Results

### 2.1. H&E Staining

**Figure 1:**
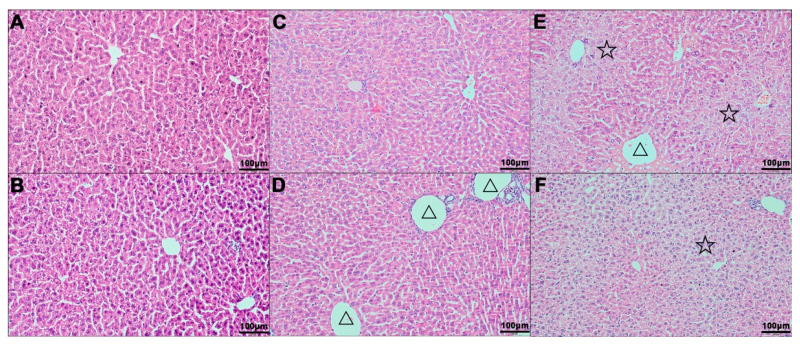
Hematoxylin and Eosin (H&E) staining of liver tissue after nutmeg exposure (200×). (**A**,**B**), control group; (**C**,**D**), low-dose group; (**E**,**F**), high-dose group.

The hepatic lobules of the mice in the control group were clearly structured, the hepatic cords and sinuses were arranged neatly and the structure of hepatocytes was completed (Figure 1A,B). In the low-dose group, the structure of hepatic lobule and the arrangement of hepatic cord were clear, the central vein of hepatic lobule was slightly dilated and the venous lumen of the portal area was also dilated slightly (△) (Figure 1C,D). In the high-dose group, the central lobule veins were slightly dilated (△) and the hepatocytes around the central lobule vein showed hypertrophy and vacuolar degeneration with unclear cell boundaries (☆) (Figure 1E,F).

### 2.2. Proteomic Pattern in the Nutmeg Exposure and Control Group

#### 2.2.1. Volcano Plots of Differentially Expressed Proteins (DEPs)

**Figure 2:**
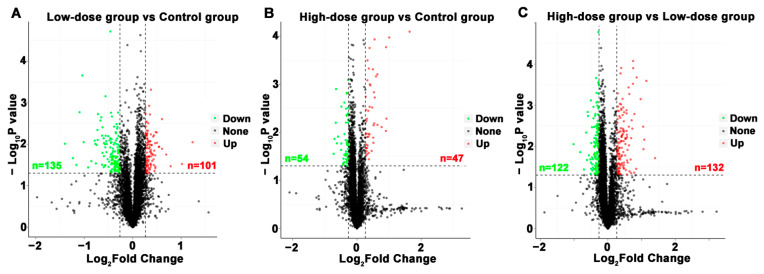
The volcano plots of differentially expressed proteins (DEPs) between different groups. (**A**) low-dose group vs. control group. (**B**) high-dose group vs. control group; (**C**) high-dose vs. low-dose group. Significant difference for the *p* value on vertical ordinate (Base 10 logarithmic transformation). Red dots represent up-regulated; green dots represent down-regulated; black dots represent no significant difference.

DEPs were displayed in a volcano graph, the black dots represent indifferent proteins, the up-regulated and down-regulated proteins were represented by red and green dots respectively (Figure 2). A total of 236 DEPs were screened between low-dose group and control group, of which 101 proteins were up-regulated and 135 were down-regulated. A total of 101 DEPs were screened between high-dose group and the control group, of which 47 were up-regulated and 54 were down-regulated. A total of 254 DEPs were screened between high-dose group and low-dose group, of which 132 proteins were up-regulated and 122 were down-regulated. The details of distinguished DEPs are listed in Tables S4–S6, Supplementary Materials.

#### 2.2.2. Cluster Analysis of DEPs

**Figure 3:**
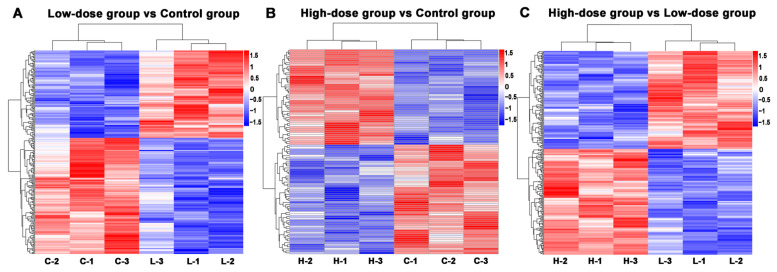
The heatmaps of differentially expressed proteins (DEPs) between different groups. (**A**) low-dose group vs. control group. (**B**) high-dose group vs. control group; (**C**) high-dose vs. low-dose group. Red represents up-regulation and blue represents down-regulation.

Cluster analysis was carried out for the relative content of DEPs in each sample and the up-regulation and down-regulation of DEPs among the comparison groups were observed in the cluster analysis of DEPs. When fold change (FC) ≥ 1.2 and *p*-value < 0.05, up-regulated expressed proteins were screened. When FC ≤ 0.83 and *p*-value < 0.05, down-regulated expressed proteins were screened. Red represents up-regulation and blue represents down-regulation (Figure 3).

#### 2.2.3. DEPs Gene Ontology (GO) Function Enrichment Analysis

**Figure 4:**
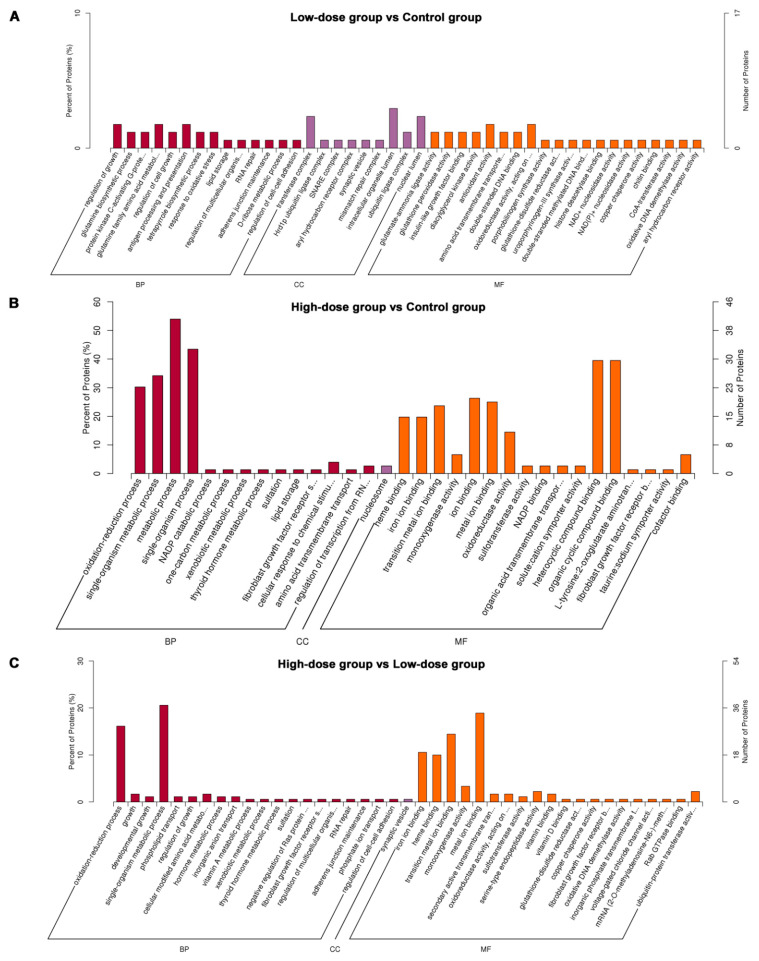
Functional enrichment of Gene Ontology (GO) annotation (Top 20) for differentially expressed proteins (DEPs). (**A**) low-dose group vs. control group. (**B**) high-dose group vs. control group; (**C**) high-dose vs. low-dose group. BP: biological Process, CC: Cellular Component, MF: Molecular Function. The left ordinate represents the number of detected differential proteins associated with the GO as a percentage of the number of differential proteins annotated by GO and the right ordinate represents the number of detected differential proteins associated with the GO.

The enrichment analysis of GO function showed the GO function entries that were significantly enriched in the DEPs compared with all the identified protein backgrounds, thereby giving the biological functions of which DEPs are significantly related to. DEPs were categorized based on three biological function terms, biological process, cell component and molecular function. GO enrichment analysis was carried out in pairs among the control group, low-dose group and high-dose group. The results were sorted according to the *p*-value from small to large, as shown in Figure 4.

Compared with the control group, the related biological processes of the screened DEPs in the low-dose group were regulation of growth, glutamine biosynthetic process, protein kinase C-activating G-protein coupled receptor signaling pathway, glutamine family amino acid metabolic process and regulation of cell growth, etc. These DEPs are mainly located in the enzyme or receptor complexes and their main molecular functions were glutamate-ammonia ligase activity, glutathione peroxidase activity, insulin-like growth factor binding, diacylglycerol kinase activity, antioxidant activity, etc. For the high- dose group, the main biological process of DEPs was oxidation-reduction process, single-organism metabolic process, metabolic process and single-organism process. In the cell component term, these proteins are associated with nucleosome and main molecular functions were mainly heme binding, iron ion binding, transition metal ion binding, monooxygenase activity, ion binding, oxidoreductase activity, sulfotransferase activity, heterocyclic compound binding and organic cyclic compound binding. In addition, the results of GO function analysis between the high-dose group and low-dose group showed similar results with those between the high-dose group and the control group.

#### 2.2.4. Enrichment of Kyoto Encyclopedia of Genes and Genomes (KEGG) Pathway Analysis

**Figure 5:**
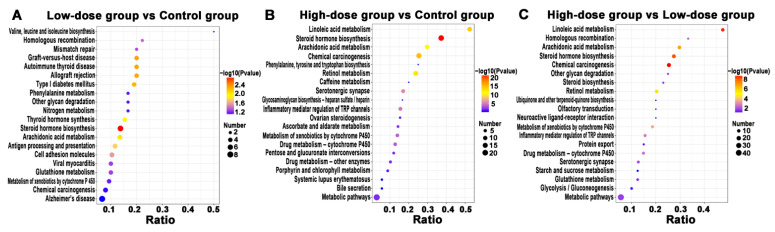
The KEGG pathway enrichment analysis of different groups. (**A**) low-dose group vs. control group. (**B**) high-dose group vs. control group; (**C**) high-dose vs. low-dose group. The abscissa represents the ratio of the number of differential proteins associated with the pathway to the number of background (all) proteins associated with the pathway. The redder the bubble represents the smaller the *p* value, the bluer the bubble represents the larger the *p* value and the larger the bubble represents the more differential proteins detected.

KEGG pathway analysis was applied for determining biochemical metabolic pathways and signal transduction pathways which involved with DEPs. According to the enrichment results, the enriched KEGG pathways were plotted (only the results of Top 20 were shown). Compared with the control group, the main enriched pathways in the low-dose group were steroid hormone biosynthesis, graft-versus-host disease, autoimmune thyroid disease, allograft rejection, type I diabetes mellitus, thyroid hormone synthesis, arachidonic acid metabolism, etc. In the comparison between high-dose group and control group, the main enriched pathways were linoleic acid metabolism, steroid hormone biosynthesis, arachidonic acid metabolism, chemical carcinogenesis and retinol metabolism etc. However, in the comparison between high-dose group and low-dose group, we found the main enriched KEGG pathways were the same as the comparison between high-dose group and control group (Figure 5 and Table S7).

#### 2.2.5. Protein–Protein Interaction (PPI) Network Analysis

**Figure 6:**
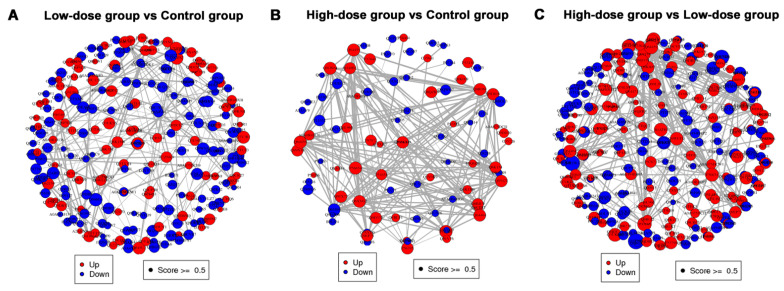
PPI networks of differentially expressed proteins (DEPs) between different groups. (**A**) low-dose group vs. control group. (**B**) high-dose group vs. control group; (**C**) high-dose vs. low-dose group. Red nodes represent up-regulated proteins and blue nodes represent down-regulated proteins.

The PPI networks of DEPs between different groups are shown in Figure 6, which revealed that most major nodal DEPs were up-regulated and the interaction between DEPs were closely in the comparison between the high-dose group and the control group or low-dose group. However, the major nodal DEPs did not show the overall change of up-regulated in the comparison between the low-dose group and the control group, and interactions between DEPs were relatively less than that between the high-dose group and the control group.

### 2.3. Biochemical Analysis of Serum Monoamine Oxidase (MAO) and Glutathione Peroxidase (GSH-Px), Malondialdehyde (MDA) and Glutathione s-Transferase (GSTs) in Liver Tissue

**Figure 7:**
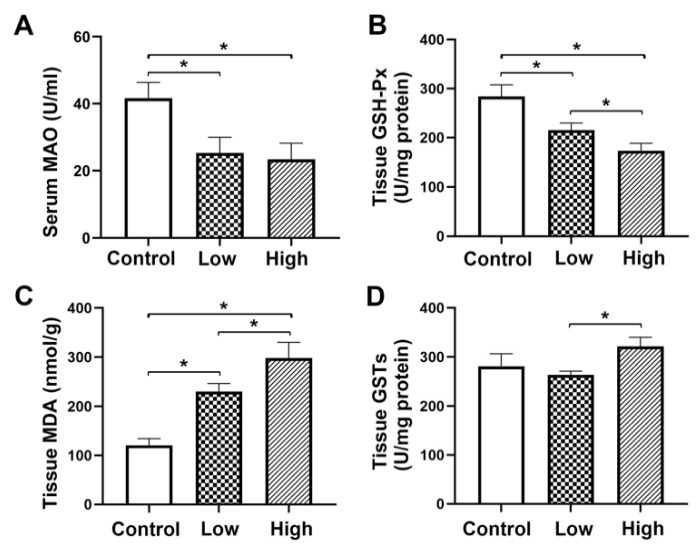
Serum monoamine oxidase (MAO) and live tissue glutathione peroxidase (GSH-Px), malondialdehyde (MDA) and glutathione s-transferase (GSTs) levels of mice after different doses exposure of nutmeg. (**A**) serum MAO level; (**B**) liver tissue GSH-Px level; (**C**) liver tissue MDA level; (**D**) liver tissue GSTs level. * *p* < 0.05. Total degrees of freedom, 17; degrees of freedom within groups, 15; degrees of freedom between groups, 2.

According to biochemical analysis, serum MAO and GSH-Px, GSTs and MDA levels in liver tissue were correlated with the dose of nutmeg exposure. Levels of serum MAO and GSH-Px in liver tissue were significantly lower in both low-dose and high-dose groups than those in the control group (*p* < 0.05) and were significantly lower in high-dose group than those in the low-dose group (*p* < 0.05) (Figure 7A,B); MDA levels in liver tissue were significantly higher in the low-dose and high-dose groups than that in the control group (*p* < 0.05) and was significantly higher in the high-dose group than that of the low-dose group (*p* < 0.05) (Figure 7C). GSTs in liver tissue revealed no significant difference in both low-dose and high-dose groups than that in the control group, but was significantly higher in the high-dose group than that in low-dose group (*p* < 0.05) (Figure 7D).

## 3. Discussion

Although nutmeg has protective and therapeutic effects, yet nutmeg abuse and poisoning were frequently reported. Currently, there are few studies on the hepatotoxicity of nutmeg. In the present study, we first explored the liver histopathological changes after different doses of nutmeg exposure and discovered that hepatocytes around the central lobule vein showed hypertrophy and vacuolar degeneration in high-dose group. Subsequently, proteomic analysis was carried out on liver tissue and it was found that cytochromes P450 (CYP450s) were up-regulated in the high-dose group, thus, led to oxidative stress. Finally, we analyzed the classical indicators of oxidative stress by enzyme-linked immunosorbent assay (ELISA) and verified the hepatic oxidative stress injury after nutmeg exposure.

Regarding the cytotoxicity, nutmeg was found to cause apoptosis, which is associated with mitochondrial membrane depolarization and Cytochrome C release [23]. Similarly, in the present study, high doses of nutmeg were found to induce elevated levels of cytochrome enzymes, including cytochromes B, C and P450. All the detected cytochrome enzymes except CYP4V and CYP2D40 showed differential expressions in the high-dose group, which were not observed in the low-dose group. Further, many nodal proteins of PPI were CYP450s, the elevated levels of which were closely associated with other DEPs. Therefore, we speculated that the up-regulated of CYP450s is the main factor of hepatotoxicity induced by nutmeg, as discussed in detail below.

CYP450s are heme-thiolate proteins, which have two main biological functions [24]. One is to metabolize exogenous substances, thereby increasing their water solubility and enabling their excretion from the body [25]. The other one is to synthesize or degrade key signaling molecules that control development and homeostasis, such as steroid hormones, fat-soluble vitamins, fatty acids and prostaglandins [24,26]. CYP450s can metabolize some drugs into active metabolites, such as free radicals, electrophilic groups and oxygen free radicals. These active metabolites can be eliminated by glutathione, however, in the case of glutathione depletion, these metabolites covalently bind macromolecules, thus resulting in lipid peroxidation, protein and DNA damage and eventually hepatocyte apoptosis and necrosis, which may be associated with the damage caused by increased hepatocyte mitochondrial permeability [27,28,29]. The liver is rich in CYP450s and is also the most important organ for drug metabolism; therefore, it is vulnerable to damage from drugs and their metabolites.

Through the analyses of DEPs GO function enrichment and KEGG enrichment, we found that the most important biological process associated with the protein expression changes in the high-dose group was the lipid metabolic process. The molecular functions of these DEPs were mainly associated with oxidation-reduction reactions and lipid binding, thereby indicating that lipid peroxidation might cause the hepatotoxicity induced by nutmeg. The top three pathways of DEPs through the comparison with high-dose group and control group were related to lipid metabolism, including linoleic acid metabolism (ratio > 0.5), steroid hormone synthesis metabolism (ratio > 0.3), arachidonic acid metabolism (ratio = 0.3). CYP450s were the main up-regulated DEPs in these three lipid metabolism pathways. Retinol was mainly stored in the liver as retinyl esters, which plays a crucial role in resisting lipid peroxidation [30,31,32]. The retinol metabolism pathway was also activated by CYP450s after high-dose nutmeg exposure.

Based on the above observations, lipid peroxidation could be determined. Similar results were also observed in the comparison between the high-dose group and the low-dose group, whereas these significant changes were only found in the high-dose group instead of the low-dose group. Lipid peroxidation is the oxidation of polyunsaturated fatty acid by free radicals in biological systems [33], which directly or indirectly affects the homeostasis and functions of cells and organs, including the immune response, fibrosis, inflammation, gene transcription, or apoptosis [34,35]. Lipid peroxidation was mainly associated with three unsaturated fatty acids—arachidonic acid, linolenic acid and linoleic acid, which can react with hydroxyl radicals or superoxide radicals [36]. Up-regulated metabolism of the front two were found in the present study, indicating the occurrence of lipid peroxidation. Biochemical analysis of MDA, which is always used as a standard for detecting lipid peroxidation, also confirmed the existence of lipid peroxidation in the present study and the degree was related to the exposure dose of nutmeg [36,37].

It was notable that CYP450s were found to participated in all the above significantly enriched pathways (*p* < 0.05) and accounted for a large proportion of DEPs in each pathway. In addition, an up-regulated level of NADPH-cytochrome P450 reductase (CPR), which transfers electrons from NADPH to CYP450s through flavin mononucleotide and can reflect the catalytic activity of CYP450s, was observed in the high-dose group, thereby exacerbating oxidative stress [38,39,40], whereas the CPR level in the low-dose group did not significantly differ from that in the control group. Therefore, we speculated that CYP450 overexpression might be induced by high-dose nutmeg, thus leading to oxidative stress.

GSH-Px is a major peroxidase enzyme that eliminates peroxides in organisms. It protects cell membranes from damage caused by peroxides and is considered the main protective system against endogenous-induced and exogenous-induced lipid peroxidation [41]. In addition, GSTs are phase II detoxification enzymes involved in maintaining cell integrity, oxidative stress and protection against DNA damage, through catalyzing glutathione conjugation to a variety of electrophilic substrates [42]. It is reported that the mechanism of drug-induced hepatotoxicity involves a lack of glutathione, thus leading to the accumulation of toxic metabolites in large quantities, mitochondrial oxidative stress and ultimately hepatic necrosis [43,44]. The increased MDA level and decreased levels of GSH-Px in liver tissue of the present study confirmed the existence of lipid peroxidation and depletion of antioxidative substances after nutmeg exposure and a dose-dependent variation. Similarly, MAO is also a classical indicator of liver damage. However, nutmeg has MAO inhibitory activity [45], which might be the reason for the decrease in serum MAO activity observed in both the high and low-dose groups. The insignificant levels of GSTs in the nutmeg-exposure group might be due to some other reasons, which are needed further study. Aspartate aminotransferase (AST) and Alanine aminotransferase (ALT) are two important indicators for evaluating liver damage. Our previous study found that levels of AST and ALT in serum were correlated with duration and dose of nutmeg exposure. Serum AST levels were significantly higher in both low and high dose group than in control groups and serum AST levels in the high-dose groups were significantly higher than those in the low-dose groups. Similarly, the serum ALT level in low dose group did not differ significantly from the control group. However, serum ALT levels in high dose groups were significantly higher than those in the control group and low-dose group. These results further demonstrated liver damage caused by nutmeg exposure [20].

In addition, selenoprotein W (SeW) is mainly found in muscles and myocardium tissues and is involved in resisting oxidative stress, removing lipid peroxides and protecting cells from oxidative damage. It is reported that its antioxidant activity depends on glutathione and SeW mRNA levels are positively correlated with glutathione content [46,47,48]. The present study showed the SeW protein was significantly down-regulated in both the high- and low-dose groups and it was down-regulated to a greater extent in the high-dose group than that in the low-dose group, thus indicating a decrease in glutathione level in the liver. This change was positively correlated with the exposure dose. In summary, the expressions of the antioxidant proteins GSH-Px and SeW in the liver tissue of mice in the two nutmeg exposure groups were down-regulated, thus suggesting that both groups experienced oxidative stress, especially for the high-dose nutmeg exposure group.

## 4. Materials and Methods

### 4.1. Chemical Reagent

Nutmeg seeds were purchased from Shenyang Medicinal Materials Market (Shenyang, China), the seeds were ground into fine powder and stored at 4 °C in a sealed plastic bag. Nutmeg powder was suspended in normal saline before each time use. MDA ELISA, MAO ELISA, Glutathione peroxidase (GSH-Px) ELISA, Glutathione S-transferase (GSTs) ELISA kits were purchased from Shanghai Enzyme-linked Biotechnology Co., Ltd. (Shanghai, China). Tandem Mass Tagging (TMT) Kits and Reagents was purchased from Thermo Fisher (Waltham, MA, USA), Bradford protein quantification kit was purchased from Beyotime (Shanghai, China). Dithiothreitol (DTT), triethyl ammonium bicarbonate buffer solution (TEAB), iodoacetamide (IAM), ammonia water and ammonium bicarbonate were purchased from Sigma (St. Louis, MO, USA). Sodium dodecyl sulfate (SDS) and urea were purchased from Sinopharm (Shanghai, China). Mass spectrometry grade pancreatin was purchased from Promega (Madison, WI, USA). Liquid chromatography-Mass spectrometry (LC-MS) grade ultrapure water, LC-MS grade acetonitrile and LC-MS grade formic acid were purchased from Thermo Fisher Chemical (Waltham, MA, USA). Acetone was purchased from Beijing Chemical Works Co., Ltd. (Beijing, China). ProteoMiner Low Abundance Protein Enrichment Kit was purchased from Bio-Rad (Hercules, CA, USA). LC-MS/MS analysis was performed by Novogene Co., Ltd. (Beijing, China).

### 4.2. Animal Model and Sample Collection

Six-week-old, specific pathogen-free (SPF) grade male Kunming mice (KM mice, originated from Swiss mice, the largest outbred group in China and widely used in pharmacology, toxicology and other fields of research) weighing 30 ± 5 g were obtained from Liaoning Changsheng Biotechnology Co., Ltd. (Shenyang, China). A total of 18 KM mice was randomly divided into control group (*n* = 6), low-dose group (*n* = 6) and high-dose group (*n* = 6). Before administration, the nutmeg powder was dissolved in normal saline, which were respectively configured as low-dose 0.05 g/mL and high-dose group 0.2 g/mL. According to the weight of each mouse, mice in the three groups were given intragastric injection for seven days and the dose is 0.2 mL/10 g for each single administration. The control group was given normal saline; the low-dose group was given 0.05 g/mL nutmeg normal saline suspension solution and the final calculated concentration was 1 g/kg; the high-dose group was given 0.2 g/mL nutmeg normal saline suspension solution, the final calculated concentration was 4 g/kg. It was reported that the LD_50_ was 5.1 g/kg and signs of abnormal behavior, including hypoactivity, unstable gait and dizziness were seen in animals given a dose of 4 g/kg or higher [4]. For preventing midway death, we chose a concentration lower than LD_50_. All the three groups were given intragastric administration with a traditional Chinese medicine mice gavage needle.

After the gavage on the 7th day, the mouse food and water were removed and all mice were fasted for 24 h before collecting blood and liver tissue as our previous methods [20]. The blood was left at room temperature for 15 min and then centrifuged at 3000 rpm for 15 min at 4 °C to obtain the serum, which was used for biochemical analysis of MAO. Mice were dissected on ice, part of the upper left lateral lobe of liver was taken and fixed in paraformaldehyde for histological analysis after washing with phosphate-buffered saline (PBS). The lower left lateral lobe of liver was taken for proteomics analysis after washing with the pre-cooled PBS and stored at −80 °C.

### 4.3. Hematoxylin and Eosin (H&E) Staining

Liver specimens from the different treatment groups were fixed in 4% paraformaldehyde for 24 h, embedded in paraffin after being washed in PBS, dehydrated in gradient alcohol, dealcoholized in xylene and then sliced into 5 µm sections. Sections were stained using standard H&E staining methods and examined by two blinded pathologists.

### 4.4. Total Protein Extraction

Liver samples (*n* = 3 in each group) were ground individually in liquid nitrogen and lysed with lysis buffer which containing 100 mM NH_4_HCO_3_ (pH 8), 8 M Urea and 0.2% SDS, followed by 5 min of ultrasonication on ice. The lysate was centrifuged at 12,000× *g* for 15 min at 4 °C and the supernatant was transferred to a clean tube. Extracts from each sample were reduced with 10 mM DTT for 1 h at 56 °C and subsequently alkylated with sufficient iodoacetamide for 1 h at room temperature in the dark. Then, samples were completely mixed with four times volume of pre-cooled acetone by vortexing and incubated at −20 °C for at least 2 h. Samples were then centrifuged and the precipitation was collected. After washing twice with cold acetone, the pellet was dissolved by dissolution buffer, which containing 0.1 M TEAB (pH 8.5) and 6 M Urea.

### 4.5. Protein Quality Test

Bovine serum albumin (BSA) standard protein solution was prepared according to the instructions of Bradford protein quantitative kit (Shanghai, China), with gradient concentration ranged from 0 to 0.5 g/L. BSA standard protein solutions and sample solutions with different dilution multiples were added into 96-well plate to fill up the volume to 20 µL, respectively. Each gradient was repeated three times. The plate was added 180 µL G250 dye solution quickly and placed at room temperature for 5 min, the absorbance at 595 nm was detected. The standard curve was drawn with the absorbance of standard protein solution and the protein concentration of the sample was calculated. A total of 20 µg of the protein sample was loaded to 12% SDS-PAGE gel electrophoresis, wherein the concentrated gel was performed at 80 V for 20 min and the separation gel was performed at 120 V for 90 min. The gel was stained by Coomassie Brilliant Blue R-250 and decolored until the bands were visualized clearly, that was shown in Figure S1.

### 4.6. TMT Labeling of Peptides

A total of 120 µg of each protein sample was taken and the volume was made up to 100 µL with dissolution buffer, 1.5 µg trypsin and 500 µL of 100 mM TEAB buffer were added, sample was mixed and digested at 37 °C for 4 h. In addition, then, 1.5 µg trypsin and CaCl2 were added, sample was digested overnight. Formic acid was mixed with digested sample, adjusted pH under 3 and centrifuged at 12,000× *g* for 5 min at room temperature. The supernatant was slowly loaded to the C18 desalting column, washed with washing buffer (0.1% formic acid, 3% acetonitrile) three times, then eluted by some elution buffer (0.1% formic acid, 70% acetonitrile). The eluents of each sample were collected and lyophilized. 100 µL of 0.1 M TEAB buffer was added to reconstitute and 41 µL of acetonitrile-dissolved TMT labeling reagent was added, sample was mixed with shaking for 2 h at room temperature. Then, the reaction was stopped by adding 8% ammonia. All labeling samples were mixed with equal volume, desalted and lyophilized.

### 4.7. Separation of Fractions

Mobile phase A (2% acetonitrile, adjusted pH to 10.0 using ammonium hydroxide) and B (98% acetonitrile) were used to develop a gradient elution. The lyophilized powder was dissolved in solution A and centrifuged at 12,000× *g* for 10 min at room temperature. The sample was fractionated using a C18 column (Waters BEH C18 4.6 × 250 mm, 5 µm) on a Rigol L3000 HPLC system, the column oven was set as 50 °C. The detail of elution gradient was shown in Table S1. The eluates were monitored at UV 214 nm, collected for a tube per minute and combined into 10 fractions finally. All fractions were dried under vacuum and then, reconstituted in 0.1% (*v*/*v*) formic acid in water.

### 4.8. LC-MS/MS Analysis

For transition library construction, shotgun proteomics analyses were performed using an EASY-nLCTM 1200 UHPLC system (Thermo Fisher, Waltham, MA, USA) coupled with a Q Exactive HF-X mass spectrometer (Thermo Fisher, Waltham, MA, USA) operating in the data-dependent acquisition (DDA) mode. A total of 1 µg sample was injected into a home-made C18 Nano-Trap column (2 cm × 75 µm, 3 µm). Peptides were separated in a home-made analytical column (15 cm × 150 µm, 1.9 µm), using a linear gradient elution as listed in Table S2. The separated peptides were analyzed by Q Exactive HF-X mass spectrometer (Thermo Fisher, Waltham, MA, USA), with ion source of Nanospray Flex™ (ESI), spray voltage of 2.3 kV and ion transport capillary temperature of 320 °C. Full scan ranges from *m*/*z* 350 to 1500 with resolution of 60,000 (at *m*/*z* 200), an automatic gain control (AGC) target value was 3 × 106 and a maximum ion injection time was 20 ms. The top 40 precursors of the highest abundant in the full scan were selected and fragmented by higher energy collisional dissociation (HCD) and analyzed in MS/MS, where resolution was 45,000 (at *m*/*z* 200) for 10 plex, the AGC target value was 5 × 104 the maximum ion injection time was 86 ms, a normalized collision energy was set as 32%, an intensity threshold was 1.2 × 105 and the dynamic exclusion parameter was 20 s.

### 4.9. The Identification and Quantitation of Protein

Raw files are directly imported into Proteome Discoverer 2.2 (PD 2.2) software for database retrieval and spectral peptide and protein quantification. The searched parameters are set as Table S3. In order to improve the quality of analysis results, the software PD 2.2 further filtered the retrieval results: Peptide Spectrum Matches (PSMs) with a credibility of more than 99% was identified PSMs. The identified protein contains at least one unique peptide. The identified PSMs and protein were retained and performed with FDR no more than 1.0%. The protein quantitation results were statistically analyzed by t-test. The proteins, whose quantitation significantly different between experimental and control groups with *p*-value < 0.05 and FC > 1.2 or < 0.833 (|log2FC| > 0.263), were defined as DEPs.

### 4.10. The Functional Analysis of Protein and DEP

GO and InterPro (IPR) functional analysis were conducted using the interproscan program against the non-redundant protein database (including Pfam, PRINTS, ProDom, SMART, ProSite, PANTHER) and the databases of COG (Clusters of Orthologous Groups) and KEGG were used to analyze the protein family and pathways. DPEs were used for Volcanic map analysis, cluster heat map analysis and enrichment analysis of GO, IPR and KEGG. The probable protein-protein interactions were predicted using the STRING-db server (http://string.embl.de/, accessed on 31 May 2020). The mass spectrometry proteomics data have been deposited to the ProteomeXchange Consortium (http://proteomecentral.proteomexchange.org, accessed on 31 May 2020) via the iProX partner repository with the dataset identifier PXD019233.

### 4.11. Validation of the Biomarkers of Oxidative Stress by ELISA

Serum was used to analyze MAO level (*n* = 6). Part of the upper left lateral lobe of liver was taken for analyzing GSH-Px, MDA and GSTs levels (*n* = 6). The specimen was homogenized with a tissue homogenizer after mixing the liver tissue with a certain amount of PBS (pH 7.4). After centrifuged for about 20 min (3000 rpm), the supernatant was collected for ELISA analysis. All steps were performed on ice and ELISA operating steps were carried out according to the manufacturer’s instructions.

### 4.12. Data Analysis

GSH-Px, MDA, GSTs and MAO levels were presented as means ± standard deviation (SD) and analyzed using IBM SPSS Statistics for Macintosh, Version 26.0 (SPSS Inc., Chicago, IL, USA). One-way analysis of variance (ANOVA) was used when appropriate and a post hoc analysis was performed if a significant difference was determined. *p* < 0.05 was considered statistically significant.

## 5. Conclusions

In conclusion, the present study employed histopathological, proteomics, biochemical methods to investigate the hepatotoxicity of nutmeg exposure and demonstrated that exposure to high doses of nutmeg might cause lipid metabolism disorders and oxidative reactive stress, subsequently antioxidants depletion, and finally, hepatocyte damage. This process might be induced primarily by CYP450 in a dose-dependent manner, which proposed potential mechanisms for hepatotoxicity after nutmeg exposure.

## Data Availability

The data presented in this study are available in article and Supplementary Materials.

## Supplementary Materials

The following are available online. Figure S1: The protein quality test by SDS-PAGE gel electrophoresis, Table S1: Peptide fraction separation liquid chromatography elution gradient table, Table S2: Liquid chromatography elution gradient table, Table S3: The analysis parameter of Proteome Discoverer 2.2, Table S4: The DEPs between the low-dose group and the control group, Table S5: The DEPs between the high-dose group and the control group, Table S6: The DEPs between the low-dose group and the high-dose group. Table S7: The major discussed DEPs after nutmeg exposure and their KEGG pathways.

## Sample Availability

Samples are available from the authors.
