# Supplementary material for: A Proteomics Study on the Mechanism of Nutmeg-Induced Hepatotoxicity
Source: Molecules. 2021 Mar 20;26(6):1748. doi: 10.3390/molecules26061748 (PMC8003901; doi:10.3390/molecules26061748)
Supplement: Supplementary file 1 [file molecules-26-01748-s001.pdf]

molecules

MDPI

Supplementary Material

A Proteomics Study on the Mechanism of Nutmeg-induced Hepatotoxicity

Wei Xia [1,†], Zhipeng Cao [1,†], Xiaoyu Zhang [1] and Lina Gao [1,*]

[1] School of Forensic Medicine, China Medical University, Shenyang 110122, P. R. China; lessen-ziale123@outlook.com (W.X.); zpcao@cmu.edu.cn (Z.C.); AjaxZhang123@126.com (X.Z.)
† The authors contributed equally to this work.
* Correspondence: lngao@cmu.edu.cn

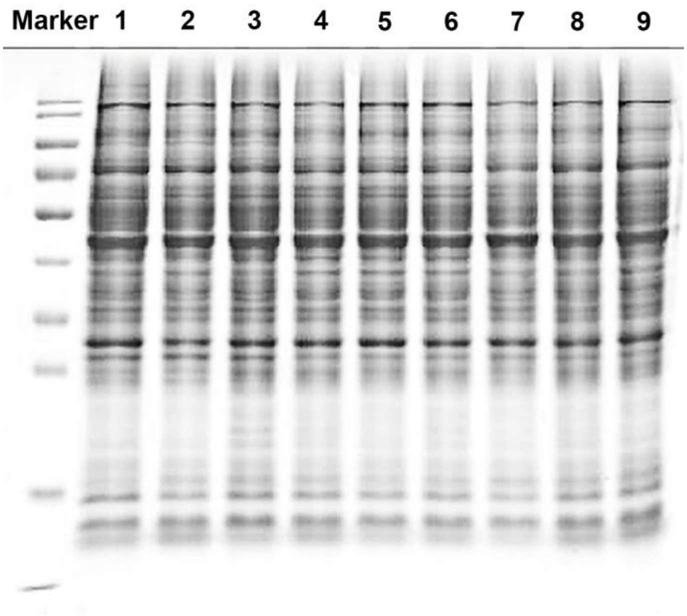

Figure S1.

Table S1. Peptide fraction separation liquid chromatography elution gradient table.

Time (min)	Flow rate (mL/min)	Mobile phase A (%)	Mobile phase B (%)
0	1	97	3
10	1	95	5
30	1	80	20
48	1	60	40
50	1	50	50
53	1	30	70
54	1	0	100

Table 2. Liquid chromatography elution gradient table.

Time (min)	Flow rate (nL/min)	Mobile phase A (%)	Mobile phase B (%)
0	600	94	6
2	600	83	17
82	600	60	40
84	600	50	50
85	600	45	55
90	600	0	100

Table S3. The analysis parameter of Proteome Discoverer 2.2.

Item	Value
Type of Quantification	Reporter Quantification (TMT)
Enzyme	Trypsin
Max.Missed Cleavage Sites	2
Precursor Mass Tolerance	10 ppm
Fragment Mass Tolerance	0.02 Da
Dynamic Modification	Oxidation/+15.995 Da (M) and TMT /+229.163 Da (K,Y)
N-Terminal Modification	Acetyl/+42.011 Da (N-Terminal) and TMT /+229.163 Da (N-Terminal)
Static Modification	Carbamidomethyl/+57.021 Da (C)

Table S4. The DEPs between the low-dose group and the control group.

Protein	Gene	Fold Change	P value	Trend
mRNA	H2-K1	0.380	0.010	down
Glutamine synthetase		0.426	0.022	down
Annexin	Anxa6	0.447	0.032	down
mRNA	H2-D1	0.467	0.002	down
Ribokinase	Rbks	0.487	0.000	down
Gamma-interferon-inducible lysosomal thiol reductase	Ifi30	0.496	0.009	down
Coiled-coil-helix-coiled-coil-helix domain-containing 10	Chchd10	0.529	0.036	down
T-cell immunoglobulin and mucin domain-containing protein 4	Timd4	0.554	0.026	down
Cytochrome c oxidase assembly factor 4 homolog, mitochondrial	COA4	0.581	0.008	down
Uncharacterized protein		0.586	0.010	down
Glycine N-acyltransferase-like protein	Gm4952	0.590	0.029	down
Isoc2a protein	Isoc2a	0.597	0.010	down
Aspartylglucosaminidase	Aga	0.603	0.005	down
Vitamin K epoxide reductase complex subunit 1	Vkorc1	0.606	0.008	down
Uncharacterized protein	Igfbp7	0.608	0.002	down
Mitochondrial intermembrane space import and assembly protein 40	Chchd4	0.630	0.003	down
Tetraspanin-9	Tspan9	0.638	0.002	down
E3 ubiquitin-protein ligase PPP1R11	Ppp1r11	0.645	0.006	down
TP53-regulated inhibitor of apoptosis 1	Triap1	0.646	0.031	down
COX assembly mitochondrial protein (Fragment)	Cmc1	0.649	0.016	down
Dynein light chain Tctex-type 3	Dynlt3	0.650	0.004	down
Coagulation factor IX	F9	0.661	0.007	down
Solute carrier family 25 member 46	Slc25a46	0.661	0.036	down
SCAN domain containing 3	Zbed5	0.662	0.019	down
Cationic amino acid transporter 2	Slc7a2	0.663	0.009	down
Protein phosphatase 1 regulatory subunit 3G	Ppp1r3g	0.670	0.033	down
BRISC and BRCA1-A complex member 2	Babam2	0.672	0.010	down
Leptin receptor gene-related protein	Leprot	0.676	0.012	down
Microtubule-associated protein	Mapt	0.677	0.032	down
MHC H-2K antigen	H2-K1	0.678	0.001	down
Macrophage migration inhibitory factor	Mif	0.680	0.006	down
ADP-ribosylation factor-related protein 1	Arfrp1	0.685	0.009	down
Alpha- and gamma-adaptin-binding protein p34	Aagab	0.686	0.016	down
Alpha-ketoglutarate-dependent dioxygenase FTO	Fto	0.690	0.010	down
Selenoprotein W	Selenow	0.698	0.023	down
Glucose-6-phosphate isomerase	Gpi1	0.704	0.006	down
Queuine tRNA-ribosyltransferase accessory subunit 2	Qtrt2	0.709	0.035	down
Protein S100-A11	S100a11	0.713	0.026	down
Lysosomal alpha-glucosidase	Gaa	0.718	0.008	down
Disintegrin and metalloproteinase domain-containing protein 17	Adam17	0.725	0.007	down
Tubulointerstitial nephritis antigen-like	Tinagl1	0.726	0.012	down
CARD domain-containing protein	Card19	0.727	0.046	down
Thymosin beta-4	Tmsb4x	0.727	0.020	down
Serum albumin	Alb	0.727	0.000	down
Eukaryotic translation initiation factor 4E type 3	Eif4e3	0.731	0.018	down
Casp7 protein	Casp7	0.732	0.009	down
NADH-ubiquinone oxidoreductase chain 3	ND3	0.733	0.044	down
Formin-binding protein 1	Fnbp1	0.734	0.002	down
DNA mismatch repair protein	Msh2	0.735	0.048	down
Protein kinase C	Prkcz	0.735	0.030	down

KH homology domain-containing protein 4	Khdc4	0.738	0.009	down
3-5 exoribonuclease 1	Eri1	0.739	0.024	down
Beta-arrestin-1	Arrb1	0.739	0.008	down
Transmembrane 7 superfamily member 3	Tm7sf3	0.741	0.047	down
[Pyruvate dehydrogenase (acetyl-transferring)] kinase isozyme 2, mitochondrial	Pdk2	0.742	0.029	down
Ribosomal_L14e domain-containing protein	Rpl14	0.743	0.002	down
MHC class II antigen D (Fragment)	H2-D1	0.745	0.021	down
Sodium- and chloride-dependent GABA transporter 2	Slc6a13	0.745	0.019	down
Transmembrane protein 11, mitochondrial	Tmem11	0.746	0.040	down
Eukaryotic translation initiation factor 6	Eif6	0.747	0.023	down
Tetraspanin-31	Tspan31	0.747	0.016	down
NHP2-like protein 1	Snu13	0.750	0.029	down
Neuroplastin	Nptn	0.751	0.030	down
Malignant T-cell-amplified sequence 1	Mcts1	0.752	0.024	down
N(G),N(G)-dimethylarginine dimethylaminohydrolase 1	Ddah1	0.753	0.013	down
Cytochrome c oxidase assembly factor 6 homolog	Coa6	0.756	0.015	down
Rho guanine nucleotide exchange factor (GEF) 26	Arhgef26	0.756	0.020	down
Dynein light chain 2, cytoplasmic	Dynll2	0.763	0.010	down
Glutathione peroxidase 3	Gpx3	0.764	0.019	down
Mannose-P-dolichol utilization defect 1	Mpdu1	0.767	0.004	down
Signal recognition particle 9 kDa protein	Srp9	0.768	0.042	down
Integrator complex subunit 10	Ints10	0.769	0.036	down
Homeodomain-only protein	Hopx	0.769	0.029	down
Cleavage stimulation factor subunit 2 tau variant	Cstf2t	0.770	0.026	down
F-box only protein 21	Fbxo21	0.772	0.026	down
DENN domain-containing protein 10	Dennd10	0.773	0.040	down
E3 ubiquitin ligase Rnf157	Rnf157	0.774	0.039	down
Tetraspanin-4	Tspan4	0.776	0.044	down
WD repeat-containing protein 41	Wdr41	0.776	0.032	down
F-box/LRR-repeat protein 20	Fbxl20	0.776	0.009	down
Glutathione reductase, mitochondrial	Gsr	0.777	0.015	down
Centrosomal protein of 170 kDa	Cep170	0.778	0.028	down
Mitochondrial import inner membrane translocase subunit Tim8 A	Timm8a1	0.784	0.022	down
Glutathione S-transferase Mu 7	Gstm7	0.785	0.031	down
RNA-binding motif, single-stranded-interacting protein 3	Rbms3	0.785	0.005	down
F-box only protein 6	Fbxo6	0.785	0.020	down
Protein canopy homolog 3	Cnpy3	0.786	0.026	down
Uncharacterized protein	Selenoo	0.787	0.020	down
Glutathione peroxidase 1	Gpx1	0.787	0.042	down
ADP-ribosyl cyclase/cyclic ADP-ribose hydrolase 1	Cd38	0.787	0.008	down
Mutant fibrillin-1	Fbn1	0.787	0.005	down
Transcriptional repressor protein YY1	Yy1	0.787	0.036	down
Ubiquinol-cytochrome-c reductase complex assembly factor 2	Uqcc2	0.790	0.029	down
Mitochondrial import inner membrane translocase subunit Tim8 B	Timm8b	0.790	0.020	down
Pleckstrin homology domain-containing family O member 2	Plekho2	0.794	0.031	down
BolA-like protein 2	Bola2	0.794	0.003	down
Delta-aminolevulinic acid dehydratase	Alad	0.797	0.044	down
Phospholipid-transporting ATPase (Fragment)	Atp11b	0.798	0.033	down
Polypyrimidine tract-binding protein 3	Ptbp3	0.799	0.010	down
Biotinidase	Btd	0.800	0.004	down
Glutamine synthetase	Glul	0.801	0.007	down
Ubiquitin carboxyl-terminal hydrolase MINDY-1	Mindy1	0.804	0.020	down

Cell adhesion molecule 1	Cadm1	0.804	0.004	down
Methyl-CpG-binding protein 2	Mecp2	0.804	0.030	down
Dolichyldiphosphatase 1	Dolpp1	0.805	0.027	down
Mycophenolic acid acyl-glucuronide esterase, mitochondrial	Abhd10	0.806	0.012	down
Transmembrane protease serine 6	Tmprss6	0.809	0.036	down
Protein tyrosine phosphatase type IVA 2	Ptp4a2	0.810	0.014	down
ATP synthase subunit epsilon, mitochondrial	Atp5f1e	0.811	0.017	down
Insulin-like growth factor-binding protein 4	Igfbp4	0.812	0.002	down
Synaptophysin-like protein 1	Sypl1	0.812	0.015	down
Uncharacterized protein	Srsf9	0.813	0.025	down
Protein C10	Grcc10	0.814	0.043	down
Pre-mRNA-splicing factor 38B	Prpf38b	0.816	0.010	down
Protein BUD31 homolog	Bud31	0.816	0.006	down
Galectin-related protein	Lgalsl	0.818	0.038	down
Ring finger and CHY zinc finger domain containing 1	Rchy1	0.818	0.040	down
Phenazine biosynthesis-like domain-containing protein 2	Pbld2	0.819	0.036	down
Serine dehydratase-like	Sdsl	0.820	0.047	down
COMM domain-containing protein 9	Commd9	0.821	0.016	down
RNA-binding protein 42	Rbm42	0.821	0.031	down
Uncharacterized protein	Prkar1a	0.823	0.014	down
THO complex subunit 3	Thoc3	0.824	0.015	down
TGc domain-containing protein	F13a1	0.825	0.048	down
Calmodulin-regulated spectrin-associated protein 3	Camsap3	0.826	0.024	down
RNA-binding protein 3	Rbm3	0.827	0.030	down
Guanidinoacetate N-methyltransferase	Gamt	0.828	0.033	down
Protein phosphatase 1 regulatory subunit 37	Ppp1r37	0.828	0.013	down
Cytochrome b-c1 complex subunit 6, mitochondrial	Uqcrh	0.829	0.011	down
Peptidyl-prolyl cis-trans isomerase G	Ppig	0.830	0.003	down
CCAAT/enhancer-binding protein zeta	Cebpz	0.830	0.036	down
RNA polymerase-associated protein CTR9 homolog	Ctr9	0.831	0.050	down
Folliculin-interacting protein 1	Fnip1	0.831	0.038	down
Phosphofurin acidic cluster sorting protein 2	Pacs2	0.832	0.033	down
Anaphase promoting complex subunit 1	Anapc1	0.833	0.041	down
Integrin alpha-3	Itga3	1.200	0.040	up
Transmembrane anterior posterior transformation protein 1	Tapt1	1.202	0.006	up
60S ribosomal protein L22-like 1	Rpl22l1	1.203	0.047	up
MAP kinase-interacting serine/threonine-protein kinase 1	Mknk1	1.204	0.002	up
E3 ubiquitin-protein ligase synoviolin	Syvn1	1.206	0.029	up
UDP-glucuronic acid decarboxylase 1	Uxs1	1.208	0.004	up
Fat storage-inducing transmembrane protein 2	Fitm2	1.209	0.003	up
Aquaporin-9	Aqp9	1.209	0.036	up
Insulin-like growth factor 2 mRNA-binding protein 3	Igf2bp3	1.210	0.047	up
Oxysterol-binding protein	Osbpl2	1.211	0.025	up
Trafficking protein particle complex subunit 2-like protein	Trappc2l	1.211	0.006	up
Mitochondrial import inner membrane translocase subunit TIM17	Timm17a	1.214	0.033	up
Required for excision 1-B domain-containing protein	Rex1bd	1.214	0.006	up
Vinexin	Sorbs3	1.215	0.009	up
Succinyl-CoA:3-ketoacid-coenzyme A transferase	Oxct1	1.217	0.049	up
DnaJ homolog subfamily C member 22	Dnajc22	1.217	0.013	up
Cytochrome P450 2C70	Cyp2c70	1.218	0.006	up
Valine--tRNA ligase, mitochondrial	Vars2	1.218	0.050	up
Uncharacterized protein	Abtb2	1.220	0.003	up
Cytochrome P450 4V2	Cyp4v2	1.220	0.032	up

Tetraspanin-8	Tspan8	1.221	0.025	up
Small cell adhesion glycoprotein	Smagp	1.221	0.006	up
Lipoprotein lipase	Lpl	1.221	0.020	up
UDP-glucuronosyltransferase	Ugt2b1	1.223	0.005	up
Importin N-terminal domain-containing protein	Ipo13	1.225	0.032	up
Phosphatidylinositol 4-kinase type 2-beta	Pi4k2b	1.225	0.016	up
GTPase Era, mitochondrial	Eral1	1.225	0.007	up
Rab22B	Rab31	1.226	0.042	up
Uncharacterized protein	Uros	1.226	0.006	up
Vacuolar protein sorting-associated protein 51 homolog	Vps51	1.227	0.006	up
Peptidyl-prolyl cis-trans isomerase FKBP11	Fkbp11	1.227	0.011	up
Syntaxin-16	Stx16	1.227	0.046	up
S-adenosyl-L-methionine-dependent tRNA 4-demethylwyosine synthase TYW1	Tyw1	1.231	0.028	up
Chloride channel protein 2	Clcn2	1.231	0.023	up
Heterogeneous nuclear ribonucleoproteins C1/C2 (Fragment)	Hnrnpc	1.233	0.005	up
Uncharacterized protein	Ddx56	1.234	0.007	up
E3 ubiquitin-protein ligase RNF139	Rnf139	1.235	0.020	up
Protein dopey-2	Dop1b	1.236	0.043	up
Ubiquitin-associated domain-containing protein 2	Ubac2	1.237	0.012	up
Structural maintenance of chromosomes flexible hinge domain-containing protein 1	Smchd1	1.242	0.031	up
Splicing factor 3b, subunit 3	Sf3b3	1.243	0.010	up
HEAT repeat-containing protein 5A	Heatr5a	1.243	0.005	up
MIF4G domain-containing protein	Mif4gd	1.244	0.033	up
Calcium signal-modulating cyclophilin ligand	Camlg	1.246	0.008	up
Replication protein A 32 kDa subunit	Rpa2	1.248	0.009	up
Leucyl-cystinyl aminopeptidase	Lnpep	1.249	0.003	up
COMM domain-containing protein 10	Commd10	1.249	0.026	up
PAM2 domain-containing protein	Paip2	1.251	0.037	up
Diacylglycerol kinase theta	Dgkq	1.254	0.009	up
Trafficking protein particle complex subunit 10	Trappc10	1.256	0.024	up
Rho-related GTP-binding protein RhoC	Rhoc	1.261	0.019	up
Probable ATP-dependent RNA helicase DDX52	Ddx52	1.262	0.020	up
Aryl hydrocarbon receptor	Ahr	1.264	0.050	up
Uncharacterized protein	Calu	1.266	0.001	up
TRMT1-like protein	Trmt1l	1.267	0.048	up
Exopolyphosphatase PRUNE1	Prune1	1.267	0.039	up
Uncharacterized protein	Ppid	1.268	0.013	up
Ras association domain-containing protein 8	Rassf8	1.270	0.020	up
Leucine carboxyl methyltransferase 1	Lcmt1	1.276	0.012	up
Receptor expression-enhancing protein	Reep4	1.277	0.010	up
Spata5 protein	Spata5	1.279	0.041	up
Proteasome assembly chaperone 4	Psmg4	1.280	0.008	up
Carbonyl reductase [NADPH] 3	Cbr3	1.280	0.015	up
Acylamino-acid-releasing enzyme (Fragment)	Apeh	1.281	0.002	up
39S ribosomal protein L42, mitochondrial	Mrpl42	1.287	0.009	up
Lysoplasmalogenase	Tmem86b	1.288	0.031	up
Estradiol 17-beta-dehydrogenase 2	Hsd17b2	1.290	0.036	up
Leucine-rich repeat-containing protein 3	Lrrc3	1.294	0.030	up
Complex I assembly factor TMEM126B, mitochondrial	Tmem126b	1.298	0.006	up
Uncharacterized protein	Mrpl40	1.299	0.014	up
Vacuolar protein sorting-associated protein 37C	Vps37c	1.299	0.000	up

mRNA-decapping enzyme 1A	Dcp1a	1.300	0.016	up
Periodic tryptophan protein 2 homolog	Pwp2	1.300	0.032	up
Son of sevenless homolog 1	Sos1	1.303	0.020	up
ATP synthase F(0) complex subunit C2, mitochondrial	Atp5mc2	1.304	0.017	up
Serine protease inhibitor A3K	Serpina3k	1.307	0.010	up
3-oxo-5-alpha-steroid 4-dehydrogenase	Srd5a1	1.315	0.010	up
Dynamin-1-like protein	Dnm1l	1.323	0.025	up
NEDD8-activating enzyme E1 regulatory subunit	Nae1	1.326	0.017	up
Ankyrin repeat domain-containing protein 40	Ankrd40	1.338	0.018	up
Alpha-1-acid glycoprotein 1	Orm1	1.342	0.007	up
Diacylglycerol kinase	Dgkd	1.349	0.023	up
Beta-galactosidase		1.354	0.032	up
Cytochrome P450 1A2	Cyp1a2	1.355	0.006	up
RNA-binding protein PNO1	Pno1	1.363	0.014	up
Monofunctional C1-tetrahydrofolate synthase, mitochondrial	Mthfd1l	1.367	0.044	up
Torsin-1B	Tor1b	1.379	0.009	up
RIKEN cDNA 2210010C04 gene	2210010C04Rik	1.380	0.019	up
Uncharacterized protein	Amfr	1.382	0.038	up
NADH-ubiquinone oxidoreductase chain 2	ND2	1.383	0.035	up
RNA helicase aquarius	Aqr	1.393	0.005	up
Cytochrome P450 2B10	Cyp2b10	1.408	0.005	up
HEAT repeat-containing protein 6	Heatr6	1.416	0.009	up
Major urinary protein 2	Mup2	1.455	0.012	up
WASH complex subunit 3	Washc3	1.462	0.011	up
p21-activated protein kinase-interacting protein 1	Pak1ip1	1.521	0.002	up
Alpha-globin	Hbat1	1.522	0.013	up
Mediator of RNA polymerase II transcription subunit 20	Med20	1.617	0.018	up
Uncharacterized protein	Cyp7b1	1.723	0.034	up
Vacuolar protein sorting-associated protein 8 homolog	Vps8	2.025	0.030	up
Podocalyxin	Podxl	2.359	0.009	up

Table S5. The DEPs between the high-dose group and the control group.

Protein	Gene	Fold Change	P value	Trend
Histone H1.4	H1-4	0.606	0.028	down
Selenoprotein W	Selenow	0.615	0.014	down
Protein kinase domain-containing protein	Stk24	0.633	0.006	down
D-glucuronyl C5-epimerase	Glce	0.637	0.001	down
NADPH-dependent 3-keto-steroid reductase Hsd3b5	Hsd3b5	0.649	0.017	down
Cationic amino acid transporter 2	Slc7a2	0.664	0.012	down
Deoxyguanosine kinase 3	Dguok	0.712	0.023	down
Glutathione peroxidase 1	Gpx1	0.713	0.017	down
Sulfotransferase	Sult2a8	0.715	0.003	down
Interferon regulatory factor 5	Irf5	0.737	0.047	down
Keratin intermediate filament 16a	K16	0.744	0.046	down
Carbonic anhydrase 1	Ca1	0.746	0.044	down
N-acetyltransferase 8	Nat8	0.747	0.035	down
Predicted gene, 20730	Gm20730	0.761	0.042	down
Anti-HIV fusion peptide monocolonal anitbody light chain vFP5.01 (Fragment)		0.762	0.029	down
Cell growth-regulating nucleolar protein	Lyar	0.762	0.002	down
Arpin	Arpin	0.765	0.011	down
Mediator of RNA polymerase II transcription subunit 12	Med12	0.781	0.031	down
Clat_adaptor_s domain-containing protein	Ap3s2	0.783	0.015	down
Chromatin target of PRMT1 protein	Chtop	0.785	0.028	down
Ring finger and CHY zinc finger domain containing 1	Rchy1	0.788	0.006	down
TYR_PHOSPHATASE_2 domain-containing protein	Ptpmt1	0.789	0.036	down
Chromobox protein homolog 8	Cbx8	0.789	0.025	down
Sulfotransferase family cytosolic 1B member 1	Sult1b1	0.792	0.003	down
Tyrosine aminotransferase	Tat	0.796	0.030	down
Uncharacterized protein	Mob3b	0.800	0.043	down
Fc receptor, IgG, low affinity IIb	Fcgr2b	0.801	0.017	down
Guanosine-3,5-bis(diphosphate) 3-pyrophosphohydrolase MESH1	Hddc3	0.801	0.004	down
MKIAA0737 protein (Fragment)	Tox4	0.802	0.002	down
Histone H2A type 2-A	Hist2h2aa1	0.802	0.005	down
Complement factor H-related protein	Gm4788	0.805	0.040	down
E3 ubiquitin-protein ligase XIAP	Xiap	0.805	0.002	down
E3 ubiquitin-protein ligase PPP1R11	Ppp1r11	0.809	0.049	down
Uncharacterized protein	Rab10	0.809	0.018	down
Sodium- and chloride-dependent taurine transporter	Slc6a6	0.811	0.018	down
Syndecan	Sdc1	0.813	0.039	down
Arf-GAP with GTPase, ANK repeat and PH domain-containing protein 3	Agap3	0.813	0.015	down
Uncharacterized protein	Tmem258	0.815	0.032	down
Unconventional myosin-Id	Myo1d	0.816	0.016	down
Leucine-rich repeat flightless-interacting protein 1	Lrrfip1	0.817	0.028	down
Nuclear factor 1 B-type	Nfib	0.823	0.022	down
Cytochrome P450, family 2, subfamily d, polypeptide 40	Cyp2d40	0.824	0.036	down
RNA/DNA-binding protein	Rnps1	0.825	0.003	down
Uncharacterized protein	Selenoo	0.826	0.033	down
Transcription elongation factor SPT4-A	Supt4h1a	0.826	0.004	down
Mid1ip1 protein	Mid1ip1	0.827	0.018	down
CMP-sialic acid transporter	Slc35a1	0.827	0.006	down
Selenide, water dikinase 2	Sephs2	0.827	0.005	down
Ankyrin repeat and SAM domain-containing protein 3	Anks3	0.828	0.035	down

Multifunctional fusion protein	Fgf1	0.828	0.001	down
Galectin-related protein	Lgalsl	0.829	0.033	down
Putative L-aspartate dehydrogenase	Aspdh	0.832	0.006	down
Rsrc1 protein (Fragment)	Rsrc1	0.832	0.019	down
Shiftless antiviral inhibitor of ribosomal frameshifting protein homolog	Shfl	0.833	0.035	down
Oxidoreductase HTATIP2	Htatip2	1.206	0.011	up
Striatin-4	Strn4	1.209	0.002	up
Serum paraoxonase/arylesterase 1	Pon1	1.209	0.021	up
Ester hydrolase C11orf54 homolog		1.215	0.003	up
Fat storage-inducing transmembrane protein 2	Fitm2	1.220	0.030	up
Cytochrome b5	Cyb5a	1.225	0.018	up
Neutral cholesterol ester hydrolase 1	Nceh1	1.225	0.022	up
Cytochrome P450 2C70	Cyp2c70	1.229	0.006	up
Cell death-inducing p53-target protein 1	Cdip1	1.230	0.025	up
Cytochrome P450, family 2, subfamily c, polypeptide 65	Cyp2c65	1.232	0.011	up
NADPH--cytochrome P450 reductase	Por	1.238	0.005	up
NADH-ubiquinone oxidoreductase chain 4	ND4	1.241	0.021	up
Complement component 6	C6	1.245	0.029	up
MKIAA0205 protein (Fragment)	Lpgat1	1.251	0.013	up
Ectonucleoside triphosphate diphosphohydrolase 5	Entpd5	1.251	0.001	up
RNA-binding protein PNO1	Pno1	1.255	0.019	up
Cytochrome P450 CYP4F15	Cyp4f15	1.255	0.000	up
UDP-glucuronosyltransferase	Ugt2b1	1.276	0.004	up
Dimethylaniline monooxygenase [N-oxide-forming] 5	Fmo5	1.279	0.012	up
26S proteasome non-ATPase regulatory subunit 4	Psmd4	1.285	0.027	up
Methyltransferase-like protein 7B	Mettl7b	1.289	0.011	up
Leukotriene-B4 omega-hydroxylase 3	Cyp4f14	1.292	0.000	up
Estradiol 17-beta-dehydrogenase 2	Hsd17b2	1.294	0.036	up
Plastin-1	Pls1	1.310	0.011	up
Solute carrier family 22 member 18	Slc22a18	1.311	0.000	up
UDP-glucuronosyltransferase	Ugt2b35	1.315	0.003	up
UDP-glucuronosyltransferase	Ugt2b34	1.318	0.001	up
Cytochrome P450 3A13	Cyp3a13	1.336	0.001	up
Pentaxin	Crp	1.372	0.024	up
Cytochrome P450 2C37	Cyp2c37	1.387	0.003	up
3-oxo-5-alpha-steroid 4-dehydrogenase	Srd5a1	1.410	0.000	up
Dynamin-1-like protein	Dnm1l	1.419	0.013	up
Pyrethroid hydrolase Ces2a	Ces2a	1.425	0.017	up
Perilipin-2	Plin2	1.430	0.007	up
Cytochrome P450 2C29	Cyp2c29	1.442	0.001	up
Proteasome subunit beta	Psmb9	1.450	0.007	up
Epoxide hydrolase 1	Ephx1	1.462	0.000	up
Cytochrome P450 2E1	Cyp2e1	1.467	0.002	up
Ubiquitin-associated domain-containing protein 2	Ubac2	1.508	0.001	up
Cytochrome P450 2C54	Cyp2c54	1.552	0.001	up
Cytochrome P450 3A11	Cyp3a11	1.556	0.007	up
H-2K(D) antigen		1.699	0.008	up
Mitochondrial 10-formyltetrahydrofolate dehydrogenase	Aldh1l2	1.868	0.009	up
Cytochrome P450 2C50	Cyp2c50	1.873	0.000	up
Cytochrome P450 1A2	Cyp1a2	1.887	0.005	up
Cytochrome P450 2C55	Cyp2c55	2.005	0.000	up
Cytochrome P450 2B10	Cyp2b10	3.107	0.000	up

Table S6. The DEPs between the low-dose group and the high-dose group.

Protein	Gene	Fold Change	P value	Trend
Podocalyxin	Podxl	0.494	0.009	down
Vacuolar protein sorting-associated protein 8 homolog	Vps8	0.567	0.014	down
InaD-like protein	Patj	0.612	0.037	down
NADPH-dependent 3-keto-steroid reductase Hsd3b5	Hsd3b5	0.641	0.005	down
Mediator of RNA polymerase II transcription subunit 20	Med20	0.652	0.022	down
p21-activated protein kinase-interacting protein 1	Pak1ip1	0.661	0.001	down
Uncharacterized protein	Amfr	0.665	0.002	down
Splicing regulatory glutamine/lysine-rich protein 1	Srek1	0.676	0.017	down
Uncharacterized protein (Fragment)	Hspa5	0.698	0.021	down
Regulation of nuclear pre-mRNA domain-containing protein 2	Rprd2	0.702	0.047	down
Uncharacterized protein	Rab10	0.705	0.001	down
3-hydroxybutyrate dehydrogenase type 2	Bdh2	0.706	0.025	down
Histone H1.4	H1-4	0.709	0.035	down
Protein dopey-2	Dop1b	0.711	0.008	down
TLC domain-containing protein 2	Tlcd2	0.721	0.018	down
Vacuolar protein sorting-associated protein 37C	Vps37c	0.722	0.003	down
Alpha-globin	Hbat1	0.726	0.027	down
Aquaporin-9	Aqp9	0.728	0.039	down
N-acetyltransferase 8	Nat8	0.733	0.020	down
Ninjurin-1	Ninj1	0.737	0.033	down
N6-adenosine-methyltransferase subunit METTL3	Mettl3	0.744	0.001	down
Ubiquitin-associated protein 2	Ubap2	0.744	0.002	down
Hdc homolog, cell cycle regulator	Heca	0.750	0.035	down
HEAT repeat-containing protein 6	Heatr6	0.753	0.010	down
Serine/threonine-protein kinase ULK3	Ulk3	0.753	0.003	down
RNA helicase aquarius	Aqr	0.753	0.008	down
NEDD8-activating enzyme E1 regulatory subunit	Nae1	0.754	0.016	down
Acp1 protein	Acp1	0.756	0.016	down
Son of sevenless homolog 1	Sos1	0.757	0.006	down
Sulfotransferase	Sult2a8	0.760	0.010	down
RNA binding protein fox-1 homolog 2	Rbfox2	0.761	0.046	down
RIKEN cDNA 2210010C04 gene	2210010C04Rik	0.764	0.036	down
CMP-sialic acid transporter	Slc35a1	0.768	0.019	down
Krt2 protein	Krt2	0.768	0.002	down
Ras association domain-containing protein 8	Rassf8	0.768	0.014	down
Leucine-rich repeat-containing protein 3	Lrrc3	0.770	0.029	down
Cytochrome P450 4V2	Cyp4v2	0.770	0.017	down
Alpha-1-antitrypsin 1-1	Serpina1a	0.770	0.045	down
Exosome complex component RRP41	Exosc4	0.772	0.008	down
Uncharacterized protein KIAA1143 homolog		0.774	0.009	down
Oxysterol-binding protein	Osbpl2	0.774	0.002	down
Hyccin	Fam126a	0.777	0.020	down
Protein FAM210A	Fam210a	0.778	0.003	down
Small cell adhesion glycoprotein	Smagp	0.779	0.005	down
Transmembrane protein 254c	Tmem254c	0.779	0.033	down
Rab3A	Rab3a	0.782	0.006	down
Probable C-mannosyltransferase DPY19L3	Dpy19l3	0.782	0.030	down
CRIB domain-containing protein	Cdc42ep4	0.783	0.039	down
Fucose-1-phosphate guanylyltransferase	Fpgt	0.784	0.018	down

Uncharacterized protein	Calu	0.785	0.025	down
Arrestin, beta 2	Arrb2	0.786	0.000	down
Sulfotransferase family cytosolic 1B member 1	Sult1b1	0.787	0.007	down
WD repeat domain phosphoinositide-interacting protein 3	Wdr45b	0.791	0.001	down
Abscission/NoCut checkpoint regulator	Zfyve19	0.792	0.008	down
E3 ubiquitin-protein ligase HECTD3	Hectd3	0.794	0.001	down
H-2 class II histocompatibility antigen, A-U beta chain		0.796	0.004	down
Monofunctional C1-tetrahydrofolate synthase, mitochondrial	Mthfd1l	0.797	0.035	down
Cytochrome P450, family 2, subfamily d, polypeptide 40	Cyp2d40	0.798	0.042	down
E3 ubiquitin-protein ligase	Nedd4l	0.799	0.015	down
WASH complex subunit 3	Washc3	0.800	0.030	down
Carbonyl reductase [NADPH] 3	Cbr3	0.800	0.028	down
39S ribosomal protein L42, mitochondrial	Mrpl42	0.800	0.004	down
Phosphoglycerate kinase (Fragment)	Pgk1	0.801	0.021	down
CMP-N-acetylneuraminate-beta-galactosamide-alpha-2,3-sialyltransferase 4	St3gal4	0.802	0.003	down
WD repeat-containing protein 18	Wdr18	0.802	0.050	down
Growth hormone-regulated TBC protein 1	Grtp1	0.803	0.015	down
Mid1ip1 protein	Mid1ip1	0.803	0.014	down
Splicing factor 3b, subunit 3	Sf3b3	0.804	0.007	down
Protein transport protein Sec16A (Fragment)	Sec16a	0.804	0.007	down
Tdrkh protein	Tdrkh	0.805	0.010	down
Multifunctional fusion protein	Fgf1	0.805	0.001	down
Replication protein A 32 kDa subunit	Rpa2	0.806	0.001	down
DNA repair protein RAD50	Rad50	0.806	0.029	down
Gap junction beta-1 protein	Gjb1	0.806	0.004	down
Sodium-dependent phosphate transporter 2	Slc20a2	0.808	0.047	down
E3 ubiquitin-protein ligase Praja-1	Pja1	0.808	0.003	down
RAS p21 protein activator 1	Rasa1	0.809	0.005	down
Nostrin	Nostrin	0.809	0.016	down
Uncharacterized protein	Abtb2	0.809	0.003	down
Trafficking protein particle complex subunit 2-like protein	Trappc2l	0.810	0.005	down
Tuftelin	Tuft1	0.810	0.045	down
Serpina1 DOM-7	DOM-7	0.810	0.029	down
Uncharacterized protein	Mrpl40	0.811	0.028	down
Uncharacterized protein	Tmem258	0.811	0.009	down
Vinexin	Sorbs3	0.811	0.002	down
Eukaryotic translation initiation factor 4H	Eif4h	0.813	0.003	down
Acyl-CoA synthetase short-chain family member 3, mitochondrial	Acss3	0.814	0.020	down
Uncharacterized protein	Ubqln1	0.815	0.009	down
Uncharacterized protein (Fragment)	Rabgap1l	0.815	0.010	down
Complement component 7	C7	0.817	0.039	down
Casein kinase I isoform gamma-3	Csnk1g3	0.818	0.002	down
Beta-mannosidase	Manba	0.819	0.011	down
Plexin B1	Plxnb1	0.819	0.045	down
Breast cancer anti-estrogen resistance protein 3	Bcar3	0.820	0.033	down
Peptidase S1 domain-containing protein	Try5	0.820	0.005	down
Glyceraldehyde-3-phosphate dehydrogenase	Gm3839	0.820	0.001	down
Probable proline--tRNA ligase, mitochondrial	Pars2	0.821	0.004	down
[F-actin]-monooxygenase MICAL3	Mical3	0.821	0.004	down
Conserved oligomeric Golgi complex subunit 3	Cog3	0.821	0.004	down
Chloride channel protein 2	Clcn2	0.821	0.004	down

Ubiquitin carboxyl-terminal hydrolase 48	Usp48	0.822	0.000	down
Calmodulin-1	Calm1	0.822	0.014	down
Structural maintenance of chromosomes flexible hinge domain-containing protein 1	Smchd1	0.823	0.011	down
Carboxypeptidase D	Cpd	0.823	0.014	down
Non-lysosomal glucosylceramidase	Gba2	0.823	0.000	down
Sin3 histone deacetylase corepressor complex component SDS3	Suds3	0.824	0.009	down
SEC22 vesicle trafficking protein homologe A (S. cerevisiae)	Sec22a	0.824	0.043	down
Unconventional myosin-Id	Myo1d	0.825	0.013	down
Uridine-cytidine kinase	Uck1	0.826	0.000	down
SWI/SNF-related matrix-associated actin-dependent regulator of chromatin subfamily E member 1	Smarce1	0.826	0.010	down
Transmembrane anterior posterior transformation protein 1	Tapt1	0.826	0.001	down
Proteasome assembly chaperone 4	Psmg4	0.827	0.018	down
Dedicator of cytokinesis 8	Dock8	0.827	0.040	down
Leucine carboxyl methyltransferase 1	Lcmt1	0.829	0.019	down
Core histone macro-H2A.2	H2afy2	0.829	0.002	down
WD repeat-containing protein 5	Wdr5	0.830	0.001	down
SH3 domain-containing protein	Bcar1	0.830	0.003	down
Cell cycle control protein 50A	Tmem30a	0.830	0.004	down
HDAg domain-containing protein	Nelfa	0.830	0.022	down
Thromboxane-A synthase	Tbxas1	0.830	0.025	down
Serine--tRNA ligase, mitochondrial	Sars2	0.830	0.034	down
5-3 exonuclease PLD3	Pld3	0.833	0.000	down
UDP-glucuronosyltransferase	Ugt2b35	1.200	0.012	up
Eukaryotic translation initiation factor 1A	Eif1a	1.202	0.010	up
Retinol dehydrogenase 13	Rdh13	1.203	0.016	up
Complement component receptor 1-like protein	Cr1l	1.206	0.027	up
Oxidoreductase HTATIP2	Htatip2	1.206	0.017	up
STE20-related kinase adapter protein beta	Stradb	1.209	0.004	up
Signal recognition particle 9 kDa protein	Srp9	1.211	0.013	up
Glutathione S-transferase theta-1	Gstt1	1.215	0.014	up
GTP-binding protein SAR1b	Sar1b	1.216	0.027	up
2-hydroxyacyl-CoA lyase 1	Hacl1	1.216	0.010	up
6-pyruvoyl tetrahydrobiopterin synthase	Pts	1.216	0.030	up
Multifunctional procollagen lysine hydroxylase and glycosyltransferase LH3	Plod3	1.219	0.043	up
Protein cereblon	Crbn	1.219	0.040	up
Ubiquitin-associated domain-containing protein 2	Ubac2	1.219	0.003	up
Acylcarnitine hydrolase	Ces2c	1.220	0.012	up
Rnase4 protein	Rnase4	1.220	0.001	up
Glutathione S-transferase Mu 1	Gstm1	1.221	0.008	up
Cytochrome b-c1 complex subunit 6, mitochondrial	Uqcrh	1.222	0.002	up
Cytochrome P450 CYP4F15	Cyp4f15	1.223	0.034	up
Ectonucleoside triphosphate diphosphohydrolase 5	Entpd5	1.225	0.005	up
Cytochrome P450 2C29	Cyp2c29	1.226	0.048	up
39S ribosomal protein L53, mitochondrial	Mrpl53	1.228	0.001	up
F-box/LRR-repeat protein 20	Fbxl20	1.230	0.015	up
Mitochondrial fission regulator 1-like	Mtfr1l	1.230	0.001	up
Phospholipid-transporting ATPase (Fragment)	Atp11b	1.233	0.047	up
Retinol-binding protein 1	Rbp1	1.236	0.012	up
Serine dehydratase-like	Sdsl	1.237	0.036	up
Malignant T-cell-amplified sequence 1	Mcts1	1.238	0.013	up

Macrophage migration inhibitory factor	Mif	1.240	0.042	up
Translocation protein SEC62	Sec62	1.243	0.044	up
Predicted gene 4788	Gm4788	1.246	0.026	up
Delta(14)-sterol reductase TM7SF2	Tm7sf2	1.246	0.007	up
Protein spinster homolog 1	Spns1	1.247	0.049	up
Synaptophysin-like protein 1	Sypl1	1.248	0.008	up
Etoposide-induced protein 2.4	Ei24	1.249	0.017	up
Transmembrane protein 33	Tmem33	1.252	0.001	up
Sigma intracellular receptor 2	Tmem97	1.255	0.027	up
Glutathione S-transferase theta-3	Gstt3	1.256	0.040	up
Uncharacterized protein	Acaa1b	1.256	0.020	up
Glucose-6-phosphatase	G6pc	1.258	0.020	up
Cytochrome P450 2E1	Cyp2e1	1.258	0.011	up
Sorting nexin-21	Snx21	1.260	0.033	up
Ubiquitin carboxyl-terminal hydrolase MINDY-1	Mindy1	1.260	0.015	up
Rho guanine nucleotide exchange factor (GEF) 26	Arhgef26	1.261	0.044	up
Cytochrome P450 3A13	Cyp3a13	1.262	0.005	up
Protein phosphatase 1 regulatory subunit 3G	Ppp1r3g	1.265	0.040	up
Leukotriene-B4 omega-hydroxylase 3	Cyp4f14	1.266	0.002	up
Uncharacterized protein	Ghdc	1.272	0.016	up
Transmembrane protease serine 6	Tmprss6	1.272	0.011	up
Phosphatidylcholine transfer protein	Pctp	1.282	0.034	up
BolA-like protein 2	Bola2	1.282	0.001	up
Alpha-ketoglutarate-dependent dioxygenase FTO	Fto	1.284	0.027	up
Dimethylaniline monooxygenase [N-oxide-forming] 5	Fmo5	1.286	0.021	up
Mycophenolic acid acyl-glucuronide esterase, mitochondrial	Abhd10	1.289	0.002	up
[Pyruvate dehydrogenase (acetyl-transferring)] kinase isozyme 2, mitochondrial	Pdk2	1.290	0.030	up
Pyrethroid hydrolase Ces2a	Ces2a	1.290	0.047	up
Cytochrome P450 3A25	Cyp3a25	1.290	0.008	up
NADH-ubiquinone oxidoreductase chain 4	ND4	1.293	0.019	up
Eukaryotic translation initiation factor 6	Eif6	1.294	0.004	up
Irgm1	OTTMUSPW KG00059374	1.298	0.048	up
Ester hydrolase C11orf54 homolog		1.298	0.000	up
F-box only protein 6	Fbxo6	1.304	0.004	up
Kelch domain-containing protein 10	Klhdc10	1.309	0.007	up
Epoxide hydrolase 1	Ephx1	1.309	0.001	up
Solute carrier family 22 member 18	Slc22a18	1.314	0.000	up
DENN domain-containing protein 10	Dennd10	1.317	0.001	up
Lymphocyte antigen 6 complex, locus G2	Ly6g2	1.318	0.001	up
Programmed cell death protein 2	Pdcd2	1.321	0.006	up
Neuroplastin	Nptn	1.324	0.017	up
Perilipin-2	Plin2	1.325	0.006	up
Transmembrane protein 11, mitochondrial	Tmem11	1.328	0.028	up
Hsp90 co-chaperone Cdc37-like 1	Cdc37l1	1.328	0.007	up
Plastin-1	Pls1	1.329	0.011	up
Disintegrin and metalloproteinase domain-containing protein 17	Adam17	1.332	0.005	up
Vitamin D-binding protein	Gc	1.336	0.017	up
Pentaxin	Crp	1.338	0.026	up
Mitochondrial import inner membrane translocase subunit Tim8 A	Timm8a1	1.340	0.005	up
Oligosaccharyltransferase complex subunit OSTC	Ostc	1.353	0.001	up

Tubulointerstitial nephritis antigen-like	Tinagl1	1.356	0.004	up
Sodium- and chloride-dependent GABA transporter 2	Slc6a13	1.356	0.002	up
Cytochrome P450 3A11	Cyp3a11	1.361	0.031	up
Vitamin K epoxide reductase complex subunit 1-like protein 1	Vkorc1l1	1.362	0.005	up
NHP2-like protein 1	Snu13	1.370	0.001	up
Uncharacterized protein	Ndufs5	1.371	0.004	up
Aldehyde dehydrogenase	Aldh3a2	1.374	0.014	up
Mannose-P-dolichol utilization defect 1	Mpdu1	1.379	0.004	up
Dynein light chain Tctex-type 3	Dynlt3	1.381	0.007	up
NADH-ubiquinone oxidoreductase chain 3	ND3	1.385	0.035	up
Cytochrome P450 2C37	Cyp2c37	1.388	0.002	up
COX assembly mitochondrial protein (Fragment)	Cmc1	1.390	0.029	up
Leptin receptor gene-related protein	Leprot	1.392	0.025	up
Cytochrome P450 1A2	Cyp1a2	1.393	0.034	up
Glutathione reductase, mitochondrial	Gsr	1.395	0.004	up
Lysosomal alpha-glucosidase	Gaa	1.397	0.001	up
Tetraspanin-31	Tspan31	1.426	0.017	up
7-dehydrocholesterol reductase	Dhcr7	1.432	0.007	up
SCAN domain containing 3	Zbed5	1.434	0.009	up
ADP-ribosylation factor-related protein 1	Arfrp1	1.436	0.006	up
Cytochrome P450 4A10	Cyp4a10	1.448	0.037	up
Mitochondrial intermembrane space import and assembly protein 40	Chchd4	1.449	0.007	up
TP53-regulated inhibitor of apoptosis 1	Triap1	1.466	0.001	up
Aspartylglucosaminidase	Aga	1.466	0.004	up
Uncharacterized protein	Igfbp7	1.468	0.002	up
Glycine N-acyltransferase-like protein	Gm4952	1.478	0.049	up
Coagulation factor IX	F9	1.478	0.005	up
HECT domain E3 ubiquitin protein ligase 4	Hectd4	1.484	0.022	up
Solute carrier family 25 member 46	Slc25a46	1.485	0.006	up
Putative bifunctional UDP-N-acetylglucosamine transferase and deubiquitinase ALG13	Alg13	1.497	0.012	up
Glucose-6-phosphate isomerase	Gpi1	1.502	0.025	up
Isoc2a protein	Isoc2a	1.530	0.000	up
BRISC and BRCA1-A complex member 2	Babam2	1.537	0.003	up
1-acylglycerol-3-phosphate O-acyltransferase ABHD5	Abhd5	1.543	0.009	up
Tetraspanin-9	Tspan9	1.554	0.001	up
Fibulin-1	Fbln1	1.558	0.041	up
Sialin	Slc17a5	1.598	0.021	up
T-cell immunoglobulin and mucin domain-containing protein 4	Timd4	1.611	0.044	up
Endonuclease/exonuclease/phosphatase family domain-containing protein 1	Eepd1	1.615	0.006	up
Vitamin K epoxide reductase complex subunit 1	Vkorc1	1.630	0.002	up
Cytochrome c oxidase assembly factor 4 homolog, mitochondrial	COA4	1.652	0.004	up
Cytochrome P450 2C55	Cyp2c55	1.675	0.000	up
RING-type domain-containing protein (Fragment)	Rnf121	1.679	0.000	up
Cytochrome P450 2C54	Cyp2c54	1.679	0.000	up
Squalene synthase	Fdft1	1.718	0.044	up
Uncharacterized protein		1.726	0.002	up
Uncharacterized protein	Psap	1.791	0.035	up
Cytochrome P450 2C50	Cyp2c50	1.808	0.000	up
Coiled-coil-helix-coiled-coil-helix domain-containing 10	Chchd10	1.961	0.001	up
Mitochondrial 10-formyltetrahydrofolate dehydrogenase	Aldh1l2	2.064	0.003	up

Glutamine synthetase		2.077	0.012	up
Glutathione S-transferase	Gm10639	2.113	0.007	up
Cytochrome P450 2B10	Cyp2b10	2.206	0.000	up
Uncharacterized protein	Grn	2.639	0.019	up

Table S7. The major discussed DEPs after nutmeg exposure and their KEGG pathways.

Protein	Group	Fold Change	P value	Trend	KEGG Pathway
CYP450 1A2	Low-dose vs Control	1.355	0.006	up	Steroid hormone biosynthesis, Caffeine metabolism, Tryptophan metabolism, Linoleic acid metabolism, Retinol metabolism, Metabolism of xenobiotics, Drug metabolism, Metabolic pathways, Chemical carcinogenesis
	High-dose vs Control	1.887	0.005	up	
	High-dose vs Low-dose	1.393	0.034	up	
CYP450 2B10	Low-dose vs Control	1.408	0.005	up	Steroid hormone biosynthesis, Arachidonic acid metabolism, Retinol metabolism, Metabolic pathways, Chemical carcinogenesis
	High-dose vs Control	3.107	0.000	up	
	High-dose vs Low-dose	2.206	0.000	up	
CYP450 2C29	High-dose vs Control	1.442	0.001	up	Steroid hormone biosynthesis, Arachidonic acid metabolism, Linoleic acid metabolism, Retinol metabolism, Metabolic pathways, Serotonergic synapse, Inflammatory mediator regulation of TRP channels, Chemical carcinogenesis
	High-dose vs Low-dose	1.226	0.048	up	
CYP450 2C37	High-dose vs Control	1.387	0.003	up	Steroid hormone biosynthesis, Arachidonic acid metabolism, Linoleic acid metabolism, Retinol metabolism, Metabolic pathways, Serotonergic synapse, Inflammatory mediator regulation of TRP channel, Chemical carcinogenesis
	High-dose vs Low-dose	1.388	0.002	up	
CYP450 2C50	High-dose vs Control	1.873	0.000	up	Steroid hormone biosynthesis, Arachidonic acid metabolism, Linoleic acid metabolism, Retinol metabolism, Metabolic pathways, Serotonergic synapse, Inflammatory mediator regulation of TRP channels, Chemical carcinogenesis
	High-dose vs Low-dose	1.808	0.000	up	
CYP450 2C54	High-dose vs Control	1.552	0.001	up	Steroid hormone biosynthesis, Arachidonic acid metabolism, Linoleic acid metabolism, Retinol metabolism, Metabolic pathways, Serotonergic synapse, Inflammatory mediator regulation of TRP channels, Chemical carcinogenesis
	High-dose vs Low-dose	1.679	0.000	up	
CYP450 2C55	High-dose vs Control	2.005	0.000	up	Steroid hormone biosynthesis, Arachidonic acid metabolism, Linoleic acid metabolism, Retinol metabolism, Metabolic pathways, Serotonergic synapse, Inflammatory mediator regulation of TRP channels, Chemical carcinogenesis
	High-dose vs Low-dose	1.675	0.000	up	
CYP450 2C70	Low-dose vs Control	1.218	0.006	up	Steroid hormone biosynthesis, Arachidonic acid metabolism, Linoleic acid metabolism, Retinol metabolism, Metabolic pathways, Serotonergic synapse, Inflammatory mediator regulation of TRP channels, Chemical carcinogenesis
	High-dose vs Control	1.229	0.006	up	
CYP450 2E1	High-dose vs Control	1.467	0.002	up	Steroid hormone biosynthesis, Arachidonic acid metabolism, Linoleic acid metabolism, Metabolism of xenobiotics, Drug metabolism, Metabolic pathways, Non-alcoholic fatty liver disease (NAFLD), Chemical carcinogenesis
	High-dose vs Low-dose	1.258	0.011	up	
CYP450 3A11	High-dose vs Control	1.556	0.007	up	Steroid hormone biosynthesis, Linoleic acid metabolism, Retinol metabolism, Metabolic pathways, Chemical car-

	High-dose vs Low-dose	1.361	0.031	up	cinogenesis
CYP450 3A13	High-dose vs Control	1.336	0.001	up	Steroid hormone biosynthesis, Linoleic acid metabolism, Retinol metabolism, Metabolic pathways, Chemical carcinogenesis
	High-dose vs Low-dose	1.262	0.005	up	
CYP450 3A25	High-dose vs Low-dose	1.290	0.008	up	Steroid hormone biosynthesis, Linoleic acid metabolism, Retinol metabolism, Metabolic pathways, Chemical carcinogenesis
CYP450 4A10	High-dose vs Low-dose	1.448	0.037	up	Fatty acid degradation, Arachidonic acid metabolism, Retinol metabolism, Metabolic pathways, PPAR signaling pathway, Vascular smooth muscle contraction, Inflammatory mediator regulation of TRP channels
CYP450 4V2	Low-dose vs Control	1.220	0.032	up	--
	High-dose vs Low-dose	0.770	0.017	down	
CYP450 CYP4F15	High-dose vs Control	1.255	0.000	up	--
	High-dose vs Low-dose	1.223	0.034	up	
CYP450, family 2, subfamily c, polypeptide 65	High-dose vs Control	1.232	0.011	up	Steroid hormone biosynthesis, Arachidonic acid metabolism, Linoleic acid metabolism, Retinol metabolism, Metabolic pathways, Serotonergic synapse, Inflammatory mediator regulation of TRP channels, Chemical carcinogenesis
CYP450, family 2, subfamily d, polypeptide 40	High-dose vs Control	0.824	0.036	down	Steroid hormone biosynthesis, Serotonergic synapse
	High-dose vs Low-dose	0.798	0.042	down	
Glutathione peroxidase 1	Low-dose vs Control	0.787	0.042	down	Glutathione metabolism, Arachidonic acid metabolism, Thyroid hormone synthesis
	High-dose vs Control	0.713	0.017	down	
Glutathione peroxidase 3	Low-dose vs Control	0.764	0.019	down	Glutathione metabolism, Arachidonic acid metabolism, Thyroid hormone synthesis
Glutathione S-transferase	High-dose vs Low-dose	2.113	0.007	up	--
Glutathione S-transferase Mu 1	High-dose vs Low-dose	1.221	0.008	up	Glutathione metabolism, Metabolism of xenobiotics, Drug metabolism, Platinum drug resistance, Pathways in cancer, Chemical carcinogenesis, Hepatocellular carcinoma, Fluid shear stress and atherosclerosis
Glutathione S-transferase Mu 7	Low-dose vs Control	0.785	0.031	down	Glutathione metabolism, Metabolism of xenobiotics, Drug metabolism, Platinum drug resistance, Pathways in cancer, Chemical carcinogenesis, Hepatocellular carcinoma, Fluid shear stress and atherosclerosis
Glutathione S-transferase theta-1	High-dose vs Low-dose	1.215	0.014	up	Glutathione metabolism, Metabolism of xenobiotics, Drug metabolism, Platinum drug resistance, Pathways in cancer, Chemical carcinogenesis, Hepatocellular carcinoma, Fluid shear stress and atherosclerosis
Glutathione S-transferase theta-3	High-dose vs Low-dose	1.256	0.040	up	Glutathione metabolism, Metabolism of xenobiotics, Drug metabolism, Platinum drug resistance, Pathways

					in cancer, Chemical carcinogenesis, Hepatocellular carcinoma, Fluid shear stress and atherosclerosis
Selenoprotein W	Low-dose vs Control	0.698	0.023	down	--
	High-dose vs Control	0.615	0.023	down	
Cytochrome b5	High-dose vs Control	1.225	0.018	up	--
Cytochrome b-c1 complex subunit 6, mitochondrial	Low-dose vs Control	0.829	0.011	down	Oxidative phosphorylation, Metabolic pathways, Cardiac muscle contraction, Non-alcoholic fatty liver disease (NAFLD), Alzheimer's disease, Parkinson's disease, Huntington's disease
	High-dose vs Low-dose	1.222	0.002	up	
Cytochrome c oxidase assembly factor 4 homolog, mitochondrial	Low-dose vs Control	0.581	0.008	down	--
	High-dose vs Low-dose	1.652	0.004	up	
Cytochrome c oxidase assembly factor 6 homolog	Low-dose vs Control	0.756	0.015	down	--
NADPH--CYP450 reductase	High-dose vs Control	1.238	0.005	up	--
Leukotriene-B4 omega-hydroxylase 3	High-dose vs Control	1.292	0.000	up	Arachidonic acid metabolism, Metabolic pathways
	High-dose vs Low-dose	1.266	0.002	up	
